# Exploring the interplay of psychiatric symptoms, antipsychotic medications, side effects, employment status, and quality of life in Chronic Schizophrenia

**DOI:** 10.1186/s12888-024-05929-3

**Published:** 2024-07-02

**Authors:** Dan-na Zhou, Xue Yang, Wen Wang, Wen-qing Jin, Yi-lang Tang, Zhi Zheng, Yanping Ren

**Affiliations:** 1 Beijing Key Laboratory of Mental Disorders, Beijing Anding Hospital, National Clinical Research Center for Mental Disorders & National Center for Mental Disorders, Capital Medical University, Beijing, China; 2 Advanced Innovation Center for Human Brain Protection, Capital Medical University, Beijing, China; 3 The First Psychiatric Hospital of Harbin, Harbin, Heilongjiang Province China; 4 Department of Psychiatry and Behavioral Sciences, Emory University School of Medicine, Atlanta, GA 30329 USA; 5 Mental Health Service Line, Atlanta VA Medical Center, Decatur, GA 30033 USA; 6 Huai’an No. 3 People’s Hospital, Huaian, Jiangsu Province China

**Keywords:** Quality of life, Schizophrenia, Clinical symptoms, Antipsychotic medication, Side effects

## Abstract

**Background:**

Many factors contribute to quality of life (QoL) in patients with schizophrenia, yet limited research examined these factors in patients in China. This cross-sectional study explores subjective QoL and its associated factors in patients.

**Methods:**

The QoL was assessed using the Schizophrenia Quality of Life Scale (SQLS). Clinical symptoms were evaluated using the Brief Psychiatric Rating Scale (BPRS) and seven factors were extracted. Patient Health Questionnaire-9 (PHQ-9), and Generalized Anxiety Disorder Scale (GAD-7) were used to assess depression and anxiety. Cognitive impairment was assessed using the Ascertain Dementia 8 (AD8). The Treatment Emergent Symptom Scale (TESS) and Rating Scale for Extrapyramidal Side Effects (RSESE) were used to evaluate the side effects of medications.

**Results:**

We recruited 270 patients (male:142,52.6%, mean age:41.9 ± 9.4 years). Positive correlations were observed between SQLS and its subdomains with the total score of BPRS, PHQ-9, GAD-7, AD8, TESS, and RSESE (all *P* < 0.005). Patients who were taking activating second-generation antipsychotics (SGAs) had lower scores on total SQLS, Motivation/ Energy domain of SQLS (SQLS-ME) as well as Symptoms/ Side effects domain of SQLS (SQLS-SS) compared to those taking non-activating SGAs (all *P* < 0.005). Multiple regression analysis showed that depressive/ anxiety symptoms and cognitive impairment had significant negative effects on QoL (*P* ≤ 0.001), while activating SGAs had a positive effect (*P* < 0.005). Blunted affect and unemployment were inversely associated with the motivation/energy domain (*P* < 0.001).

**Conclusion:**

Our findings emphasize the important role of depression/anxiety symptoms and cognitive impairment in the QoL of patients with chronic schizophrenia. Activating SGAs and employment may improve the QoL of these individuals.

**Trial registration:**

This protocol was registered at chictr.org.cn (Identifier: ChiCTR2100043537).

## Background

Schizophrenia, a chronic and severe mental disorder, intricately impacts various aspects of patients’ lives, including cognition, emotion, and social functioning. A primary objective in the treatment of schizophrenia is to improve the quality of life (QoL) of patients. QoL reflects patients’ subjective well-being and satisfaction with their life circumstances. Notably, a low level of QoL is often a risk factor for relapses [1], underscoring the importance of understanding related factors to improve patient rehabilitation and prevent relapses.

Prior studies have identified multiple factors that negatively influence quality of life in patients with schizophrenia. Clinical symptoms are consistently reported as one of the predictors of QoL in schizophrenia, potentially affecting the ability of patients to cope with daily challenges, engage in meaningful activities, and maintain social relationships. The impact of positive, negative, and general psychopathology symptoms on the QoL in schizophrenia has been substantiated by previous research [2, 3]. A meta-analysis of 56 studies by Eack and Newhill highlights the negative association between general psychopathology (such as anxiety, depression and poor insight) and QoL across all indicators. Additionally, negative symptoms, such as apathy, anhedonia, and social withdrawal was also found to be associated with poor QoL [4]. Mao et al. found that positive symptoms, particularly impulsivity/hostility, adversely affected physical and psychological aspects of QoL [5], while hallucinations and delusions had a weaker and less consistent relationship with QoL. Notably, affect symptoms including depressive symptoms were related to QoL in schizophrenia patients attract more attention in recent years [5–7] which needs further study. Furthermore, impaired neurocognition has been linked to reduced QoL [8]. A previous study involving 79 patients with schizophrenia suggested that cognitive impairment and severe negative symptoms were strong predictors of quality of life [9]. Several studies suggested that cognitive deficits were independently associated with poor QoL in older patients with schizophrenia [8, 10, 11]. However, other studies suggested that there was no correlation between positive symptoms, negative symptoms [6], and cognitive impairment with QoL [6, 12]. These inconsistent results may be attributed to the heterogeneity of the patient population, the sample size, and the variety of assessment tools used to measure symptoms and QoL, including SQLS, 36-Item Short Form Survey (SF-36), the World Health Organization Quality of Life (WHOQOL)-Brief Form (BREF) [5, 6, 13].

Antipsychotic medications, the mainstay of pharmacological treatment for schizophrenia, have a dual influence on QoL. Their efficacy on symptoms may improve QoL [14] but their side effects may negatively impact QoL because of uncomfortable feelings [15]. Furthermore, side effects can also reduce QoL by reducing adherence to medications. Interestingly, some partial dopamine agonists (such as aripiprazole and amisulpride) had activating effects [16–19] and aripiprazole was found to have significantly greater improvements for the Positive and Negative Syndrome Scale (PANSS) negative subscale scores and Montgomery-Asberg Depression Rating Scale (MADRS) total score than haloperidol [20]. A recent study also showed that aripiprazole could improve interest and activity [21] and QoL [22] in patients with major depressive disorder, but the extent and direction of these effects in schizophrenia are not well understood.

The relationship between clinical symptoms, antipsychotic medications, and side effects on QoL in schizophrenia is a complex and understudied area. Previous studies have examined how clinical symptoms and side effects affect social functioning, but the relative contribution of factors and the role of medication type are unclear. Moreover, the impact of psychiatric symptoms, antipsychotic medications, and side effects on QoL is not static or uniform across patients and settings and may interact with each other and with other factors, such as social support, unmet needs, coping skills, and circumstances [23, 24].

Despite the progress in research, there are still significant gaps in our understanding of the multifaceted factors associated with QoL in schizophrenia. A robust, large sample study involving a broad range of variables is essential to elucidate the independent effect of each predictor, control for confounding variables, and clarify the roles of various factors associated with QoL. Such insights are crucial for developing targeted interventions to improve QoL in these patients.

In this study, we sought to build a comprehensive model of predictors of QoL in patients with schizophrenia using data from a clinical study conducted across 13 centers in China. Utilizing structural equation modeling, we concurrently examined the effects of six broad groups of clinical factors previously identified as potential predictors of QoL in this population. These categories include (1) severity of psychiatry symptoms, (2) severity of depression and anxiety symptoms, (3) severity of cognitive impairment, (4) type of psychotropic medications, and (5) sociodemographic characteristics. Based on existing literature and our preliminary analyses, we hypothesized that QoL in patients with chronic schizophrenia would be significantly associated with depressive/anxiety symptoms and cognitive impairment. Additionally, we posited that the type of psychotropic medications, particularly activating agents like amisulpride and aripiprazole, might also influence QoL positively.

## Methods

The study sample consisted of inpatients and outpatients diagnosed with schizophrenia from 18 psychiatric hospitals across 13 provinces and regions of China. The hospitals were selected based on their region and hospital level. Inclusion criteria were as follows: 18–65 years of old, with a diagnosis of schizophrenia according to the Diagnostic and Statistical Manual for Mental Disorders. Fourth Edition, Text Revision (DSM-IV, TR) [25]. Exclusion criteria included severe medical disorders, such as organic mental disability, dementia, blindness, deaf-muteness, or other severe physical disabilities; comorbidities with other mental disorders including mental retardation, dementia, and other severe cognitive impairment; active alcohol/substance use disorders.

This study was approved by the Ethics Committee of Beijing Anding Hospital, Capital Medical University (approval number 2020-research-45). All participants or their family members were required to provide written informed consent before participating.

### Data collection

Data was collected by face-to-face interviews using a locally developed questionnaire covering socio-demographic characteristics (e.g. gender, age, education, marital status, etc.) and clinical characteristics (e.g. age of onset, course of illness, hospitalization frequency). Information on antipsychotic medications was also collected, categorizing them into first-generation antipsychotics (FGAs) and second-generation antipsychotics (SGAs). SGAs were further divided into clozapine, activating SGAs (amisulpride and aripiprazole), non-activating SGAs (risperidone, olanzapine, quetiapine, ziprasidone, paliperidone, and lurasidone). Other medication groups included antidepressants, benzodiazepines, mood stabilizers, and others.). We calculated dose equivalents based on defined daily doses (DDDs) presented by the World Health Organization’s Collaborative Center for Drug Statistics Methodology [26]. Doses equivalent to 1 mg olanzapine, and we divided doses equivalent to olanzapine into three groups (≤ 10 mg group, 10-20 mg group and > 20 mg group).

#### QoL assessment

The QoL was assessed using the Schizophrenia Quality of Life Scale (SQLS) which has 30 self-assessment items across three domains: (1) The Psychosocial (PS) domain focuses on emotion expression and interpersonal communication function, including 15 items (I feel angry, I worry about my future, I feel lonely, I feel hopeless, I feel very jumpy and edgy, I take things people say the wrong way, I find it hard to concentrate, I find it difficult to mix with people, I feel down and depressed, I feel very mixed up and unsure of myself, my feelings go up and down, I am concerned that I won’t get better, 1 worry about things, I feel that people tend to avoid me, I get upset thinking about the past); (2) The Motivation/energy (ME) domain, which assesses motivation and energy levels, includes 7 items (I lack the energy to do things, I can’t be bothered to do things, I am able to carry out my day to day activities, I take part in enjoyable activities, I like to plan ahead, I tend to stay at home, I feel that I can cope); (3) The Symptoms/Side effects (SS) domain comprises 8 items assessing various symptoms and side effects (I am bothered by my shaking/trembling, I feel unsteady walking, I am troubled by a dry mouth, my muscle get stiff, my vision is blurred, my sleep is disturbed, I get muscle twitches, I get dizzy spells) [27]. Each item was rated on a 5-point scale from 0 to 4 (0: never, 1: rarely, 2: sometimes, 3: often, 4: always). The total score ranged from 0 (the best status) to 100 (the worst status).

### Clinical assessment

Psychiatric symptoms were assessed using the Brief Psychiatric Rating Scale (BPRS) [28] The BPRS has 18 items that are rated on a 7-point scale from 1 (not present) to 7 (most severe), with a total score ranging from 18 to 126. We selected some BPRS items that correspond to the three dimensions of psychopathology (Psychoticism-grandiosity, suspiciousness, unusual thought content, hallucinatory behavior; Disorganization-conceptual disorganization, mannerisms/ posturing; Negative symptoms-blunted affect) defined by DSM-IV [29] as remission criteria.

We also analyzed the effect of seven core symptoms that indicate remission on QoL, instead of categorizing psychotic symptoms into positive and negative ones. Depression and anxiety symptoms were evaluated using the Patient Health Questionnaire-9 (PHQ-9) and Generalized Anxiety Disorder Scale (GAD-7) self-report scales. The PHQ-9 has nin items with a total score from 0 to 36 and the GAD-7 has seven items with a total score from 0 to 21 [30, 31]. A higher score indicated a worse condition.

#### Cognitive function assessment

The Ascertain Dementia 8 (AD8) was used to assess patients’ cognitive and functional abilities [32]. It included eight items to assess memory, orientation, and judgment [33]. The total score ranges from 0 to 8 and the lower score means better cognition function, while a score 2 or more suggests possible cognition impairment. It has good sensitivity (72–91%) and specificity (67–78%) [34], and it has been used in various healthcare settings and patient populations.

#### Side effects assessment

To assess the side effects of medication therapy, we used the Treatment Emergent Symptom Scale (TESS) [35] and the Rating Scale for Extrapyramidal Side Effects (RSESE) [36]. TESS is a 35-item scale that measures the severity and management of side effects. The scale of severity ranged from 0 (no) to 4 (severe), the scale of management ranged from 0 (no) to 6 (stop treatment), with a total score of 0-350. RSESE included 10 items on a scale from 0 (no) to 4 (consistent exists) of each item to evaluate the extrapyramidal reaction, with a total score of 0 to 40 [37]. The higher scores indicate more severe side effects. We added RSESE to account for the side effects of complex medication regimens used in our study.

#### Analysis

All study data were analyzed using SPSS software (version 25.0, SPSS Inc., Armonk, NY, United States). Continuous data were reported as mean ± standard deviation (SD), and group comparisons were performed using independent-sample *t*-tests. Categorical data were described using composition proportions. We assessed relationships between clinical characteristics (BPRS, PHQ-9, GAD-7, TESS, and RSESE), cognitive functions (AD8), the total and dimensional scores of SQLS using Pearson’s correlation analysis for normally distributed data, and Spearman’s rank correlation for non-normally distributed data. To explore potential predictors of QoL, multinomial logistic regression models were used and bivariate regressions were performed to select predictors for the multiple regression model. Our conceptual model included six groups of potential predictors: (1) severity of psychiatry symptoms, (2) severity of depressive and anxiety symptoms, (3) severity of cognitive impairment, (4) type of psychotropic medications, (5) severity of side effects, and (6) sociodemographic characteristics. For regression analysis, standardized Beta (β) coefficients with 95% confidence interval (CI) were computed to assess the level of association and statistical significance.

### Data quality control

Before the study began, all data collectors and supervisors received training on data collection tools. A pre-test was conducted with schizophrenia patients (not involved in this study) to test the validity and reliability of the questionnaire and other instruments. The interrater reliability (IRR) for BPRS was 0.83, for TESS it was 0.88, and for the RESSE it was 0.79. The supervisors and the principal investigator (Y-pR) also reviewed the data for completeness and consistency every day during data collection.

## Results

A total of 270 patients with schizophrenia, either as outpatients or inpatients, were enrolled in our study. Two patients failed to complete TESS and RSESE, and four patients missed information on medication treatment. Therefore, the final analysis comprised 264 patients who completed all required assessments of the study.

### Socia-demographic and clinical characteristics comparisons of SQLS and its domains

Comparisons revealed significant differences between the patients with and without a family history of mental illness in total SQLS scores (40.8 ± 13.7 vs. 34.5 ± 16.5, t=-2.35, *P* = 0.021) and psychosocial (PS) scores (33.9 ± 14.8 vs. 27.6 ± 16.4, *t* = 2.29, *P* = 0.023). Patients with an age of onset above 40 years old exhibited significantly lower SQLS scores (24.5 ± 14.9 vs. 36.2 ± 16.2, *t* = 2.10, *P* = 0.037) and motivation/energy (ME) scores (36.5 ± 16.1 vs. 47.6 ± 16.7, *t* = 2.33, *P* = 0.021) than those with an onset of age below 40. The PS scores differed significantly between patients with an educational level below high school and those with high school and above (27.7 ± 15.7 vs. 33.0 ± 18.5, *t*=-2.02, *P* = 0.044), between those with monthly household income per capita below 1000RMB (China Yuan) and those above 1000RMB (29.7 ± 16.2 vs. 24.9 ± 16.2, *t* = 2.01, *P* = 0.046). The ME scores were significantly lower in the employed patients compared to unemployed patients (31.2 ± 12.7 vs. 47.9 ± 16.5, *t* = 4.08, *P* < 0.001). Patients with an illness duration above 10 years exhibited a significantly higher symptoms/side effects (SS) score (18.3 ± 16.2 vs. 13.5 ± 12.1, *t*=-2.20, *P* = 0.029). No significant differences in the total SQLS scores and its domain scores in other variable groups (See Table 1).

**Table 1:** The relationships between demographic characteristics, clinical features and quality of life measures in patients with chronic schizophrenia

Variables *N*(270)	Variable categories	Frequency *n* ( %)	Total SQLS			PS			ME			SS		
			M ± SD	t	*P*	M ± SD	t	*P*	M ± SD	t	*P*	M ± SD	t	*P*
Gender	Male	142(52.6)	34.9 ± 17.0	-0.60	0.548	27.8 ± 16.6	-0.86	0.390	46.4 ± 16.2	-0.47	0.640	17.2 ± 16.7	0.31	0.754
	Female	128(47.4)	36.1 ± 15.3			29.5 ± 16.0			47.4 ± 17.0			16.6 ± 13.7		
Age	≤ 33 years	65(24.1)	35.1 ± 15.4	-0.31	0.754	28.5 ± 15.8	0.14	0.893	47.5 ± 16.3	0.43	0.666	17.8 ± 15.6	-1.64	0.103
	> 34 years	182(67.4)	35.0 ± 17.2			28.3 ± 16.8			47.6 ± 18.9			14.2 ± 14.1		
Marital	Singles	142(32.6)	35.6 ± 16.5	0.11	0.878	29.3 ± 16.7	0.51	0.445	45.8 ± 16.8	-1.03	0.303	16.9 ± 14.8	-0.03	0.979
status	Married	128(47.4)	35.4 ± 16.0			27.9 ± 16.8			48.0 ± 16.8			16.9 ± 16.0		
Education	high school	223(82.6)	38.0 ± 17.8	-1.13	0.259	27.7 ± 15.7	-2.02	0.044	47.0 ± 16.8	0.27	0.787	17.0 ± 15.6	0.28	0.780
	Above high school	48(17.4)	35.1 ± 15.9			33.0 ± 18.5			44.5 ± 17.7			16.3 ± 14.2		
Employed	yes	17(6.3)	33.1 ± 14.9	0.62	0.526	27.2 ± 16.7	-0.90	0.678	31.2 ± 12.7	4.08	0.000	20.1 ± 16.9	-1.02	0.307
	no	253(93.7)	35.7 ± 16.3			29.2 ± 16.2			47.9 ± 16.5			16.6 ± 15.2		
With chronic medical disorder														
	yes	33(12.2)	35.1 ± 16.3	-1.08	0.282	31.3 ± 14.0	0.98	0.328	48.2 ± 16.9	-0.49	0.623	19.1 ± 12.2	-0.83	0.407
	no	237(87.8)	38.4 ± 15.8			28.2 ± 16.6			46.7 ± 16.8			16.6 ± 15.7		
Family history														
	yes	43(17.8)	40.8 ± 13.7	-2.35	0.021	33.9 ± 14.8	2.29	0.023	50.3 ± 15.6	-1.47	0.143	19.4 ± 13.6	-1.08	0.282
	no	227(82.2)	34.5 ± 16.5			27.6 ± 16.4			46.2 ± 16.9			16.4 ± 15.6		
Age of onset														
	< 40 years	253(93.7)	36.2 ± 16.2	2.10	0.037	29.1 ± 16.4	1.52	0.129	47.6 ± 16.7	2.33	0.021	17.2 ± 15.5	0.88	0.378
	≥ 40 years	17(6.3)	24.5 ± 14.9			20.1 ± 15.1			36.5 ± 16.1			12.8 ± 11.4		
Duration of illness														
	≤ 10 years	83(30.8)	33.2 ± 16.1	-1.55	0.123	27.2 ± 16.7	-0.90	0.371	45.8 ± 17.3	-0.70	0.485	13.5 ± 12.1	-2.20	0.029
	> 10 years	187(69.2)	36.5 ± 16.1			29.2 ± 16.2			47.4 ± 16.6			18.3 ± 16.2		
Hospitalizations														
	≤ 3 times	126(46.7)	37.1 ± 16.5	1.21	0.244	29.9 ± 16.6	0.96	0.338	48.0 ± 15.9	0.68	0.498	16.0 ± 15.4	1.16	0.249
	>3 times	144(53.3)	34.7 ± 16.1			27.9 ± 16.4			46.6 ± 17.7			18.4 ± 15.3		
Per capita household income, mo														
	≤ 1000RMB	209(77.4)	36.4 ± 16.0	1.76	0.080	29.7 ± 16.2	2.01	0.046	46.8 ± 17.2	0.31	0.758	17.4 ± 15.5	1.07	0.284
	> 1000 RMB	61(22.6)	32.3 ± 16.7			24.9 ± 16.2			46.1 ± 16.3			14.9 ± 14.7		

SQLS: Quality of Life Scale, PS: Psychosocial, ME: Motivation/ Energy, SS: Symptoms/ Side effects, RMB: China Yuan

Data are reported as Mean ± SD, unless indicated otherwise

### Effects of antipsychotic medications on SQLS and its domains

We compared the effects of different types of medications on SQLS and found no group differences among patients who were on FGAs (6%), SGAs (88%), FGAs + SGAs (4.5%), and unmedicated (1.5%) groups. After regrouping medications based on whether they have activating properties, we found that compared to those who were on non-activating medications, patients on activating SGAs (amisulpride and aripiprazole) had a significantly lower total SQLS score (37.2 ± 16.5 vs. 30.7 ± 14.4, *t* = 2.80, *P* = 0.005), ME score (48.5 ± 16.5 vs. 40.3 ± 17.5, *t* = 3.45, *P* = 0.001) and SS score (18.2 ± 15.9 vs. 13.5 ± 12.9, *t* = 2.01, *P* = 0.045). ME score (49.8 ± 16.5 vs. 45.0 ± 17.1, *t*=-2.14, *P* = 0.033) was higher while the SS score (13.9 ± 11.9 vs. 18.7 ± 15.4, *t* = 2.35, *P* = 0.02) were significantly lower in the patients who were on clozapine compared to those who were on non- clozapine group. See Table 2.

**Table 2:** The relationships between antipsychotic medication characteristics and quality of life measures in patients with chronic schizophrenia

Medication (*N* = 266)			Frequency *n*( %)	Total SQLS			FS			ME			SS		
				M ± SD	F	*p*	M ± SD	F	*P*	M ± SD	F	*P*	M ± SD	F	*P*
Type of AP	FGAs		16(6.0)	33.8 ± 15.2	1.25	0.291	28.2 ± 14.3	1.54	0.204	47.9 ± 21.0	0.33	0.904	13.6 ± 12.7	1.95	0.123
	SGAs		234(88.0)	35.8 ± 16.5			29.3 ± 16.5			46.6 ± 16.8			17.1 ± 15.6		
	FGAs + SGAs		12(4.5)	39.6 ± 14.1			28.3 ± 14.1			49.5 ± 14.3			25.1 ± 13.9		
	Unmedicated		4(1.5)	22.0 ± 7.0			11.8 ± 7.0			44.8 ± 18.0			7.0 ± 6.2		
Clozapine		yes	86(32.3)	35.0 ± 14.0	0.41	0.686	27.8 ± 14.0	0.75	0.454	49.8 ± 16.5	-2.14	0.033	13.9 ± 11.9	2.35	0.020
		no	180(67.7)	35.9 ± 17.3			29.4 ± 17.3			45.0 ± 17.1			18.7 ± 15.4		
Activating		yes	64(24.1)	30.7 ± 14.4	2.80	0.005	25.5 ± 15.0	1.90	0.058	40.3 ± 17.5	3.45	0.001	13.5 ± 12.9	2.01	0.045
SGAs		no	202(57.9)	37.2 ± 16.5			30.0 ± 16.6			48.5 ± 16.5			18.2 ± 15.9		
Other SGAs		Yes	157(59.0)	36.0 ± 17.8	-0.52	0.601	29.4 ± 17.8	-0.61	0.543	45.8 ± 17.3	0.89	0.377	18.2 ± 16.1	-1.31	0.192
		no	109(41.0)	35.0 ± 13.8			28.1 ± 13.9			47.7 ± 16.8			15.6 ± 14.2		
Others		Yes	55(20.7)	36.1 ± 17.6	-0.27	0.791	30.9 ± 17.7	-1.01	0.315	42.9 ± 16.9	1.80	0.073	18.1 ± 15.1	-0.55	0.582
		no	211(79.3)	35.5 ± 15.9			28.4 ± 15.9			47.5 ± 17.0			16.8 ± 15.4		
Combination		Monotherapy	168(64.1)	36.5 ± 16.4	0.96	0.338	29.3 ± 16.5	0.31	0.759	47.6 ± 16.6	1.33	0.186	18.4 ± 16.2	1.54	0.126
		Drug combination	94(35.9)	34.5 ± 16.2			28.7 ± 15.9			44.6 ± 18.2			15.2 ± 13.9		
DDD		≤ 10 mg	128(48.9)	36.4 ± 17.4	1.18	0.318	29.5 ± 17.7	1.56	0.199	47.2 ± 17.1	0.24	0.870	18.0 ± 16.3	1.06	0.356
		10-20 mg	109(41.6)	35.6 ± 14.9			28.7 ± 14.2			46.4 ± 17.0			17.1 ± 14.5		
		> 20 mg	25(9.5)	33.6 ± 16.6			29.2 ± 17.2			44.2 ± 18.9			13.8 ± 13.2		

AP: Antipsychotic; FGAs: first-generation antipsychotics; SGAs: second-generation antipsychotics; DDD: defined daily doses

Data are reported as Mean ± SD, unless indicated otherwise

### Clinical symptoms and side effects associated with QoL and its domains

Spearman’s correlation analyses revealed significant positive correlations between total SQLS scores and various clinical symptoms. Specifically, grandiosity (*r* = 0.22, *P* < 0.001), suspiciousness (*r* = 0.37, *P* < 0.001), unusual thought content (*r* = 0.35, *P* < 0.001), hallucinatory behavior (*r* = 0.44, *P* < 0.001), conceptual disorganization (*r* = 0.35, *P* < 0.001), mannerisms/ posturing (*r* = 0.26, *P* < 0.001), and blunted affect (*r* = 0.22, *P* < 0.001) all were positively associated with SQLS. There were significant correlations between the total score of SQLS with PHQ-9 (*r* = 0.64, *P* < 0.001) and GAD-7 (*r* = 0.57, *P* < 0.001).

**Fig. 1:**
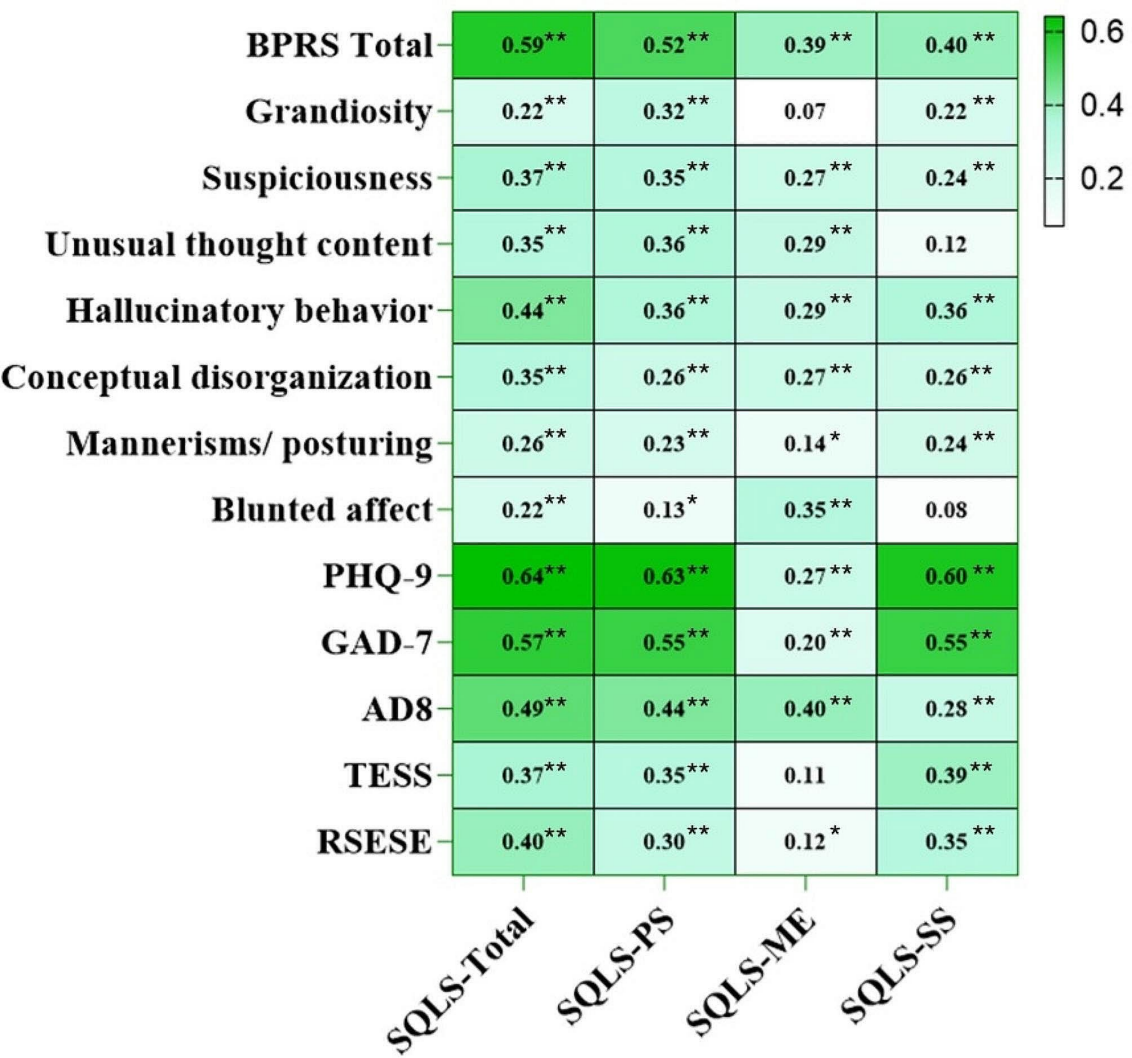
Correlations between clinical characteristics and SQLS scores, sub-domains scores in patients with schizophrenia. *Abbreviations* SQLS, Quality of Life Scale; BPRS, Brief Psychiatric Rating Scale; PHQ-9, Patient Health Questionnaire-9; GAD-7, Generalized Anxiety Disorder Scale; AD8, The Ascertain Dementia; TESS, Treatment Emergent Symptom Scale; RSESE, Rating Scale for Extrapyramidal Side Effects. Results are Pearson’s or Spearman’s correlation coefficient. *P < 0.05, **P < 0.01.

The mean score of cognition score (AD8) was 2.7 ± 2.3. 169 patients (62.59%) scored 2 or higher. A significant correlation was observed between the total score of SQLS with AD-8 (*r* = 0.49, *P* < 0.001). Additionally, there was a significant correlation between the total SQLS score and TESS (*r* = 0.37, *P* < 0.001), and RSESE (*r* = 0.40, *P* < 0.001). The dimensional scores, including PS, ME, and SS, exhibited similar correlations with the severity of clinical symptoms and side effects. See Fig. 1.

### Bivariate regressions and multivariate regression analysis of influential factors on SQLS and its domains

We conducted bivariate regression analyses to examine the effects of factors on SQLS and its domains. The results suggested that the total score of SQLS was significantly associated with family history, age of onset, activating SGAs, BPRS total score and item scores, PHQ-9, GAD-7, TESS, and RSESE(all *P* < 0.05). The dimensional score of PS was significantly associated with education level, family history, Per capita household income, BPRS total score and item scores, PHQ-9, GAD-7, TESS, and RSESE(all *P* < 0.05). The dimensional score of ME was significantly associated with employed, clozapine, activating SGAs, BPRS total score, and item scores except for grandiosity, PHQ-9, GAD-7, and RSESE(all *P* < 0.05). The dimensional score of SS was significantly associated with duration of illness, clozapine, activating SGAs, BPRS total score, and item scores except for unusual thought content and blunted affect, PHQ-9, GAD-7, TESS, and RSESE(all *P* < 0.05). See Table 3.

**Table 3:** Bivariate regression analysis between factors and the quality of life in patients with chronic schizophrenia

variable	Total SQLS			PS			ME			SS		
	β(95%CI)	*t*	*P*	β(95%CI)	*t*	*P*	β(95%CI)	*t*	*P*	β(95%CI)	*t*	*P*
Education (high school)	0.07(-2.09-8.25)	1.17	0.242	0.12(0.49–10.40)	1.99	**0.048**	-0.01(-5.84-5.00)	-0.15	0.88	-0.02(-5.75-4.31)	-0.28	0.778
Family history of mental illness (yes)	0.14(1.04–11.58)	2.36	**0.019**	0.14(0.96–11.67)	2.32	**0.021**	0.09(-1.24-9.86)	1.53	0.127	0.08(-2.09-8.54)	1.19	0.234
Age of onset (< 40 years)	-0.15(-18.02–2.13)	-2.50	**0.013**	-0.13(-16.96–0.47)	-1.08	0.058	-0.13(-10.84–1.19)	-1.25	0.135	-0.09(-14.09-2.42)	-1.39	0.165
Duration of illness (≤ 10 years)	0.09(-0.96-0.75)	1.53	0.127	0.07(-2.03-6.80)	1.07	0.266	0.04(-2.89-6.16)	0.71	0.48	0.14(0.26–8.90)	2.01	**0.038**
Employed (yes)	-0.03(-5.76-3.58)	-0.44	0.646	0.03(-3.49-5.88)	0.50	0.617	-0.24(-14.35–4.84)	-3.98	**< 0.001**	0.07(-2.17-7.40)	1.08	0.282
Percapitahousehold income, mo, (≤ 1000RMB)	-0.11(-8.77–0.49)	-1.76	0.081	-0.12(-5.97–0.90)	-2.01	**0.046**	-0.02(-5.64-4.11)	-0.31	0.758	-0.07(-7.28-2.14)	-1.07	0.284
On clozapine (yes)	-0.03(-5.07-3.34)	-0.41	0.686	-0.05(-5.84-2.62)	-0.75	0.45	0.13(0.38–9.13)	2.14	**0.033**	-0.15(-8.93–0.78)	-2.35	**0.020**
On activating SGAs (yes)	-0.17(-10.99–1.92)	-2.80	**0.005**	-0.12(-9.07-0.16)	-1.90	0.058	-0.21(-13.00–3.55)	-3.45	**0.010**	-0.13(-9.21–0.10)	-2.01	**0.045**
BPRS total score	0.52(0.55–0.83)	9.86	**< 0.001**	0.45(0.46–0.75)	8.11	**< 0.001**	0.35(0.33–0.64)	6.04	**< 0.001**	0.40(0.36–0.65)	6.84	**< 0.001**
Grandiosity	0.19(1.30–5.57)	3.17	**0.002**	0.16(0.68–5.01)	2.59	**0.010**	0.07(-0.93-3.61)	1.16	0.246	0.22(1.62–5.75)	3.51	**0.001**
Suspiciousnes	0.36(2.84–5.47)	6.22	**< 0.001**	0.31(2.26–5.01)	5.20	**< 0.001**	0.28(1.99–4.82)	4.74	**< 0.001**	0.24(1.28–3.97)	3.85	**< 0.001**
Unusual thought contents	0.31(2.49–5.50)	5.24	**< 0.001**	0.27(2.05–5.16)	4.56	**< 0.001**	0.28(2.26–5.43)	4.78	**< 0.001**	0.12(-0.11-2.99)	1.83	0.069
Hallucinatory behavior	0.38(3.58–6.55)	6.72	**< 0.001**	0.31(2.53–5.61)	5.20	**< 0.001**	0.27(2.09–5.33)	4.51	**< 0.001**	0.36(3.11–6.12)	6.05	**< 0.001**
Conceptual disorganization	0.31(2.81–6.08)	5.35	**< 0.001**	2.33(1.66–5.09)	3.88	**< 0.001**	0.26(2.18–5.66)	4.43	**< 0.001**	0.26(1.87–5.20)	4.13	**< 0.001**
Mannerisms/ posturing	0.25(2.79–7.66)	4.23	**< 0.001**	0.21(1.89–6.84)	3.47	**< 0.001**	0.16(0.82–6.01)	2.59	**< 0.001**	0.24(2.31–6.99)	3.92	**< 0.001**
Blunted affect	0.24(1.55–4.48)	4.04	**< 0.001**	0.13(0.17–3.23)	2.19	**0.029**	0.36(3.21–6.16)	6.24	**< 0.001**	0.08(-0.53-2.45)	1.28	0.203
PHQ-9 score	0.645(11.11–14.80)	11.43	**< 0.001**	0.63(10.75–14.54)	13.13	**< 0.001**	0.27(3.33–8.20)	4.67	**< 0.001**	0.60(9.31–13.08)	11.70	**< 0.001**
GAD-7 score	0.55(10.14–14.71)	10.63	**< 0.001**	0.51(9.25–13.98)	9.66	**< 0.001**	0.20(2.03–7.62)	3.39	**0.001**	0.55(9.49-14.00)	10.25	**< 0.001**
AD8 score	0.45(2.37–3.87)	8.16	**< 0.001**	0.40(2.05–3.62)	7.10	**< 0.001**	0.40(2.15–3.76)	7.21	**< 0.001**	0.28(1.07–2.69)	4.58	**< 0.001**
TESS score	0.37(0.37–0.58)	13.80	**< 0.001**	0.34(0.28–0.55)	5.74	**< 0.001**	0.11(-0.02-0.32)	1.23	0.126	0.39(0.31–0.58)	5.70	**< 0.001**
RSESE score	0.32(1.09–2.34)	10.70	**< 0.001**	0.27(0.84–2.11)	4.56	**< 0.001**	0.13(0.08–1.45)	2.20	**0.029**	0.35(1.25–2.57)	6.59	**< 0.001**

SQLS: Quality of Life Scale, PS: Psychosocial, ME: Motivation/ Energy, SS: Symptoms/ Side effects, RMB: China Yuan (6.86 RMBs ≈ 1US dollar), SGAs: second-generation antipsychotics, BPRS: Brief Psychiatric Rating Scale, PHQ-9: Patient Health Questionnaire-9, GAD-7: Generalized Anxiety Disorder Scale, AD8: Ascertain Dementia 8, TESS: Treatment Emergent Symptom Scale, RSESE: Rating Scale for Extrapyramidal Side Effects

Data are reported as Mean ± SD, unless indicated otherwise

**Fig. 2:**
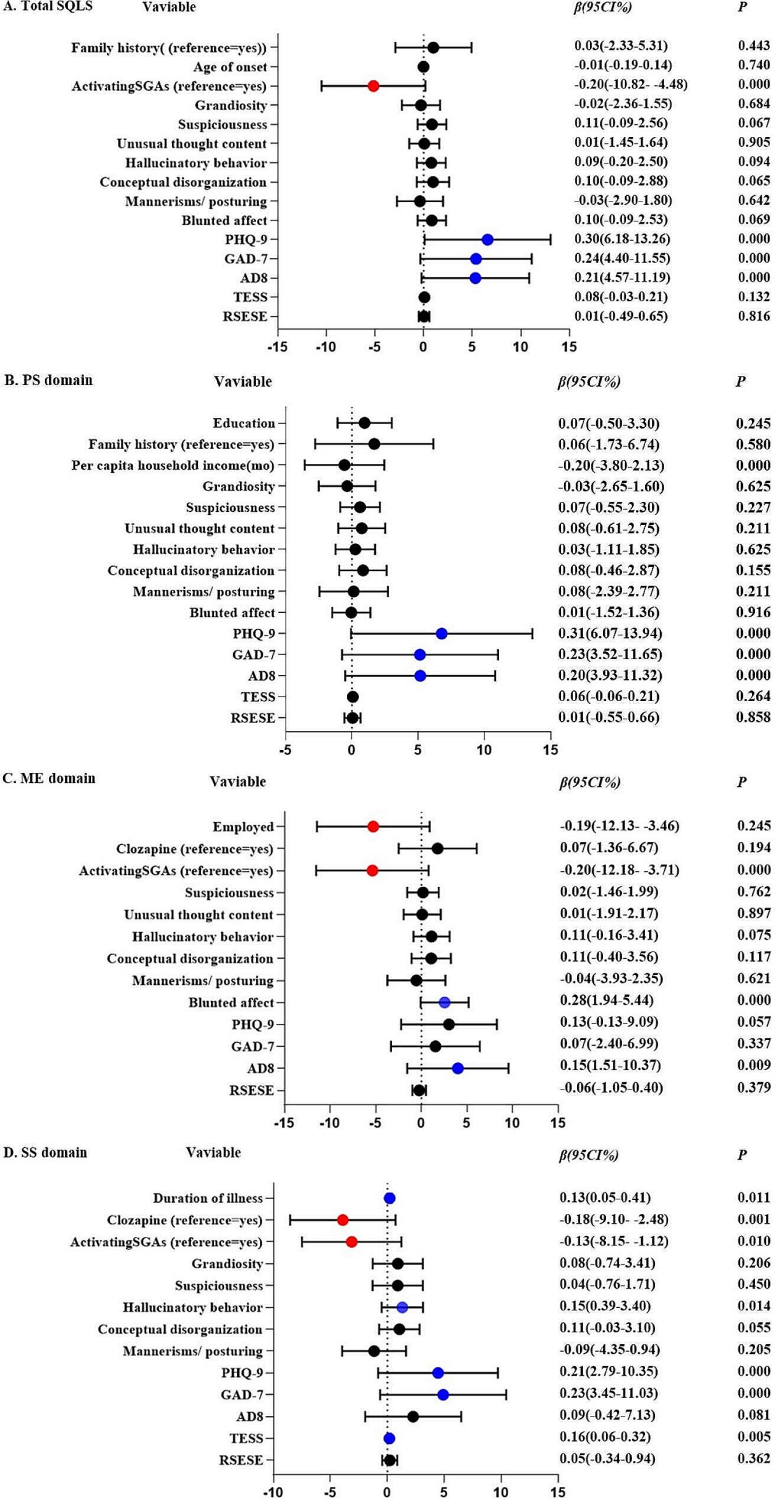
Multivariate regression analysis of factors associated with QoL and its domains in patients with schizophrenia. (**A**) Effect of factors on the total QoL. (**B**) Effect of factors on the domain of PS. (**C**) Effect of factors on the domain of ME. (**D**) Effect of factors on the domain of SS. *Abbreviations* PS: Psychosocial, ME: Motivation/ Energy, SS: Symptoms/ Side effects. Results are Unstandardized Beta (β) coefficients with 95% confidence interval (CI)

Furthermore, we conducted a multivariate linear regression analysis to examine the effects of multiple factors on SQLS and its domain including socio-demographic variables, clinical symptoms, cognition, antipsychotic features, and side effects. We found that the SQLS total scores were significantly associated with depression (PHQ-9, *β* = 0.30, *P* < 0.001), anxiety (GAD-7, *β* = 0.24, *P* < 0.001), and cognitive impairment (AD8, *β* = 0.21, *P* < 0.001), while they were inversely associated with the use of activating SGAs (*β*=-0.20, *P* < 0.001). Similar associations were observed between PS, PHQ-9 (*β* = 0.31, *P* < 0.001), GAD-7(*β* = 0.23, *P* < 0.001) and AD8 scored (*β* = 0.20, *P* < 0.001). ME scores positively correlated with both bunted affect (*β* = 0.28, *P* < 0.001) and AD8 scores (*β* = 0.15, *P* < 0.001), while negatively associated with employment (*β*=-0.19, *P* < 0.001) and the use of activating SGAs (*β*=-0.20, *P* < 0.001). SS scores were positively correlated with the duration of the illness (*β* = 0.13, *P* = 0.011), hallucinatory behavior (*β* = 0.15, *P* = 0.014), depression (*β* = 0.21, *P* = 0.001), anxiety (*β* = 0.23, *P* < 0.001), and side effects (*β* = 0.16, *P* = 0.005) but negatively associated with the use of clozapine (*β*=-0.18, *P* = 0.001) and activating SGAs (*β*=-0.13, *P* = 0.010) (See Fig. 2).

## Discussion

This study represents as one of the few to investigate the multifaced impact of clinical symptoms, cognition, psychiatric medications, and side effects on QoL in patients with chronic schizophrenia. Our findings shed light on key factors influencing the QoL of these patients and offer insights into potential intervention and improvement.

### Depressive/anxiety symptoms and cognitive impairment

consistent with previous research on QoL in patients with schizophrenia, our study underscores the significant influence of depressive/anxiety symptoms and cognitive impairment on the overall QoL and subdomains. Numerous previous studies have documented the association in patients with schizophrenia including first-episode schizophrenia and chronic schizophrenia [5, 6, 13, 38, 39]. The prominence of depressive cognitions, including self-deprecation and feelings of hopelessness, suggests a need for targeted intervention to address these symptoms [5]. Treating depressive symptoms may emerge as a crucial clinical strategy for the improvement of subjective QoL in patients with schizophrenia [6]. Our study did not find significant differences in SQLS scores and its domains in patients who were on antidepressants, mood stabilizers, or benzodiazepines, potentially due to the relatively small sample sizes in the subgroups. Furthermore, the symptoms of depression/ anxiety in chronic schizophrenia may be multifactorial, including psychosocial, biological factors, medication side effects [40], etc., and they are often difficult to distinguish from negative symptoms [41]. Further exploration with larger cohorts is warranted to elucidate the nuanced effects of clinical symptoms on QoL in schizophrenia patients.

### Cognitive impairment’s impact on functional deficits

Our study uniquely contributes to the understanding of the effects of cognitive impairment on QoL, PS, and ME. The findings on the effects of cognitive impairment on QoL in the existing literature were inconsistent, in part due to the use of different assessment tools for cognition in different studies and sample differences, which suggest the need for comprehensive evaluation. Ishii et al. used the Mini-Mental State Examination-Japanese (MMSE-J) to analyze cognitive impairments of hospitalized patients with schizophrenia and reported that cognition impairment was not significantly associated with the Qol [6]. In our study, cognitive impairment was assessed using AD8, a more sensitive for mild cognition impairment and is more suitable for outpatient screening [33]. We showed that cognitive impairment was negatively correlated with total QoL, PS, and ME domains. This is consistent with other studies [10, 42]. Therefore, assessment and interventions for cognitive impairment in patients with chronic schizophrenia should also be given due attention.

### Negative symptoms, unemployment, and Motivation/Energy Domain

Our study also found significant links between the ME domain and blunted affect and unemployment. Previous research has suggested that negative symptoms such as social withdrawal can directly affect QoL in patients with schizophrenia [4], while other studies have reported weak or no correlation between positive/negative symptoms and QoL [4, 43]. These inconsistent results may be due to the broad measurement method. Instead of using the total score of the negative symptoms in PANSS, which did not correlate with QoL [5, 6], we analyzed the association between QoL and seven core symptoms that reflect remission of disease, which was more specific to the assessment of negative symptoms. We found that blunted affect (such as amotivation and diminished expression) correlated with overall health level, consistent with previous findings [44]. In line with previous findings, employment was positively associated with the motivation and energy of QoL. A recent study reported that improvement in employment status had a stronger impact on mental health than improvement in education or income in patients with mental illness [45]. Another study also showed that occupational balance can improve the QoL in patients with schizophrenia [7]. This highlights the importance of vocational training and supervised employment to improve QoL in patients with schizophrenia, and any psychiatric rehabilitative programs should incorporate employment-related components.

### Activating second-generation antipsychotics (SGAs) and QoL

A novel and somewhat surprising finding from our study is that the use of activating SGAs was associated with better QoL, as reflected in lower total QoL, ME, and SS domains. These medications may exhibit efficacy in alleviating deficit symptoms and improving negative symptoms, promoting QoL [14, 16, 44, 46]. Previous studies also reported that aripiprazole and amisulpride were superior to lurasidone, cariprazine, olanzapine, and FGAs in improving QoL [16, 47, 48]. Shared decision-making processes with patients may consider these interventions to enhance QoL effectively.

### Side effects and clozapine’s impact

Our study showed that the SS domain was negatively associated with side effects in patients with chronic schizophrenia. Two main explanations have been proposed: Firstly, side effects such as oversedation, anticholinergic, and weight gain, may directly affect QoL [15, 49–51]. Secondly, side effects may decrease medication adherence [52] and increase the risk of relapse, and poor insight leads to inaccurate self-reporting of QoL [53]. To enhance the QoL of patients, it is recommended that acute antipsychotic drug therapy should be based on side effect profiles [54]. Consistent with previous research, we found that the use of clozapine was positively associated with QoL in the SS domain. Treatment with clozapine led to improvement in core symptoms of schizophrenia [55] and resulted in fewer extrapyramidal symptoms compared with FGAs, which also improved QoL [56]. Therefore, a comprehensive consideration of the interplay between psychotic symptoms, cognitive function, antipsychotic drugs, and side effects is crucial in treatment decision-making for patients with schizophrenia.

## Limitations

While this study offers valuable insights into the factors associated with QoL in schizophrenia patients, it is important to recognize a few limitations. The cross-sectional design restricts the ability to infer causality between the variables studied and QoL. Additionally, due to the cross-sectional design, we do not have data on the duration of different treatments, which could also affect symptoms and QoL. Longitudinal research would be advantageous for examining these relationships over time. Furthermore, the sample was largely drawn from psychiatric hospitals within specific areas, potentially affecting the applicability of the results to wider populations or different healthcare contexts. While a comprehensive range of variables was examined, additional factors that could influence QoL in schizophrenia were not explored in this study. The use of self-reported data for certain variables, including QoL and cognitive function, might have introduced bias due to the possibility of self-assessment inaccuracies. Finally, although AD8 has been widely used for the early detection of dementia in different settings and populations, its reliability and validity in assessing cognitive impairments in patients with schizophrenia has not been well tested. Future studies should address these limitations to enhance our understanding of QoL in this patient group.

## Conclusions

In conclusion, we found that depression, anxiety, and cognitive impairment emerge as key factors related to poorer QOL in patients with chronic schizophrenia. The use of activating SGAs, such as aripiprazole and amisulpride, and addressing negative symptoms may offer potential benefits. The comprehensive consideration of psychotic symptoms, cognitive function, antipsychotic drugs, and side effects is crucial for guiding the treatment of schizophrenia and enhancing patients’ QoL.

## Data Availability

Data are available from the corresponding authors upon reasonable request.

## Abbreviations

QoL: Quality of life
SQLS: Quality of life scale
PS: Psychosocial
ME: Motivation/energy
SS: Symptoms/side effects
RMB: China Yuan
FGAs: First-generation antipsychotics
SGAs: Second-generation antipsychotics
DDDs: Defined daily doses
BPRS: Brief psychiatric rating scale
DSM-IV-TR: Diagnostic and Statistical Manual for Mental Disorders. Fourth Edition, Text Revision
PHQ-9: Patient health questionnaire-9
GAD-7: Generalized anxiety disorder scale
AD8: Ascertain Dementia 8
TESS: Treatment emergent symptom scale
RSESE: Rating scale for extrapyramidal side effects
SD: Standard deviation
CI: Confidence interval
n (%): Composition ratio
SF-36: 36-Item Short Form Survey
WHOQOL-BREF: The World Health Organization Quality of Life (WHOQOL)-Brief Form (BREF)
PANSS: Positive and negative syndrome scale
MADRS: Montgomery-Asberg depression rating scale
